# SMART: physical activity and cerebral metabolism in older people: study protocol for a randomised controlled trial

**DOI:** 10.1186/s13063-015-0662-9

**Published:** 2015-04-11

**Authors:** Johannes Fleckenstein, Silke Matura, Tobias Engeroff, Eszter Füzéki, Valentina A Tesky, Ulrich Pilatus, Elke Hattingen, Ralf Deichmann, Lutz Vogt, Winfried Banzer, Johannes Pantel

**Affiliations:** Department of Sports Medicine, Institute of Sports Sciences, Goethe University, Ginnheimer Landstrasse 39, Frankfurt am Main, 60487 Germany; Institute of General Practice, Goethe University, Frankfurt/Main, Germany; Institute of Neuroradiology, Goethe University Hospital Frankfurt, Frankfurt, Germany; Brain Imaging Centre, Frankfurt/Main, Germany

**Keywords:** Magnetic resonance spectroscopic imaging, Aerobic exercise training, Cognitive impairment, Older adults, Psychometric tests, Cognition, Dementia, Prevention

## Abstract

**Background:**

Physical activity exerts a variety of long-term health benefits in older adults. In particular, it is assumed to be a protective factor against cognitive decline and dementia.

**Methods/design:**

Randomised controlled assessor blinded 2-armed trial (n = 60) to explore the exercise- induced neuroprotective and metabolic effects on the brain in cognitively healthy older adults. Participants (age ≥ 65), recruited within the setting of assisted living facilities and newspaper advertisements are allocated to a 12-week individualised aerobic exercise programme intervention or a 12-week waiting control group. Total follow-up is 24 weeks. The main outcome is the change in cerebral metabolism as assessed with Magnetic Resonance Spectroscopic Imaging reflecting changes of cerebral N-acetyl-aspartate and of markers of neuronal energy reserve. Imaging also measures changes in cortical grey matter volume. Secondary outcomes include a broad range of psychometric (cognition) and movement-related parameters such as nutrition, history of physical activity, history of pain and functional diagnostics. Participants are allocated to either the intervention or control group using a computer-generated randomisation sequence. The exercise physiologist in charge of training opens sealed and opaque envelopes and informs participants about group allocation. For organisational reasons, he schedules the participants for upcoming assessments and exercise in groups of five. All assessors and study personal other than exercise physiologists are blinded.

**Discussion:**

Magnetic Resonance Spectroscopic Imaging gives a deeper insight into mechanisms of exercise-induced changes in brain metabolism. As follow-up lasts for 6 months, this study is able to explore the mid-term cerebral metabolic effects of physical activity assuming that an individually tailored aerobic ergometer training has the potential to counteract brain ageing.

**Trial registration:**

NCT02343029 (clinicaltrials.gov; 12 January 2015).

## Background

Cognitive impairment and dementia is a pressing health care issue in older people (as described previously [1]). However, physical activity is thought to be beneficial in altering the trajectory of cognitive decline in older adults [2-4]. Although there is still considerable uncertainty concerning the appropriate type, intensity and duration of physical activity, there is evidence that regular low- to medium-level exercise leads to a 35% risk reduction in cognitive decline in people aged 65 and above compared to inactive older people [5].

The potential mechanisms behind the protective effects of physical activity on cognitive function are thought to be multidimensional. It has been suggested that aerobic exercise renders the brain more efficient, plastic, and adaptive, which leads to improved memory and executive function [2,6,7]. In brief, mechanisms comprise positive functional changes in haemodynamic activity [4], neurogenesis and neural cell proliferation [8] with newly formed neurons being integrated functionally into neural networks [9] and synaptic plasticity [10]. On a molecular basis, exercise differentially regulates synaptic proteins associated with the function of the brain-derived neurotrophic factor (BDNF) [11]. Further, exercise is assumed to impact the production of insulin-like growth factor 1 (IGF-1) [12].

As a correlate of neural plasticity, neuroimaging trials show physical activity to induce structural changes in the human brain [13-16]. A reduced loss of grey matter volume in the brain has been associated with higher levels of cardiorespiratory fitness (for review: [17]). These changes in grey matter volume, especially in the hippocampus have been linked to an improved memory function [13], which could potentially delay the onset of dementia. The influence of physical activity on neural plasticity is closely linked or even dependent on various aspects of brain energy metabolism. Exercise up-regulates multiple proteins within the hippocampus that have a defined role in energy metabolism, comprising enzymes involved in glucose catabolism, ATP synthesis and glutamate turnover [18].

In addition to the structural changes in the ageing brain, other cofactors have been attributed to cognitive decline. Ito *et al*. showed not only older age, but also lower mental health well-being, daytime sleepiness, pain and lower instrumental activities of daily living to be significant correlates of memory complaints [19]. Chronic pain seems to play an important role as related states of mood such as depression, fatigue and pain catastrophising have been associated with subjective cognitive complaints [20]. This has been shown to remarkably impair cognitive performance [21].

We conduct a randomised controlled assessor blinded 2-armed trial to investigate the effects of a 12-week individualised aerobic exercise programme on cerebral metabolism, as well as grey matter volume and cognitive functioning in cognitively healthy older adults, when compared to a waiting control group. To address the complexity of several other biopsychosocial cofactors of brain metabolism, we take every-life confounders such as nutrition, lifetime physical activity or pain experience into account.

## Methods/design

### Design of the study

This is a randomised controlled partially blinded 2-armed trial to evaluate the effects of a 12-week individualised aerobic exercise programme on the cerebral metabolism in cognitively healthy older adults, when compared to a waiting control group. The study has been approved by the Ethics Committee of the Goethe University of Frankfurt am Main, Germany (reference 107/13) and is in agreement with the Declaration of Helsinki (Version Fortaleza 2012). Trial registration is NCT02343029 (clinicaltrials.gov).

**Figure 1:**
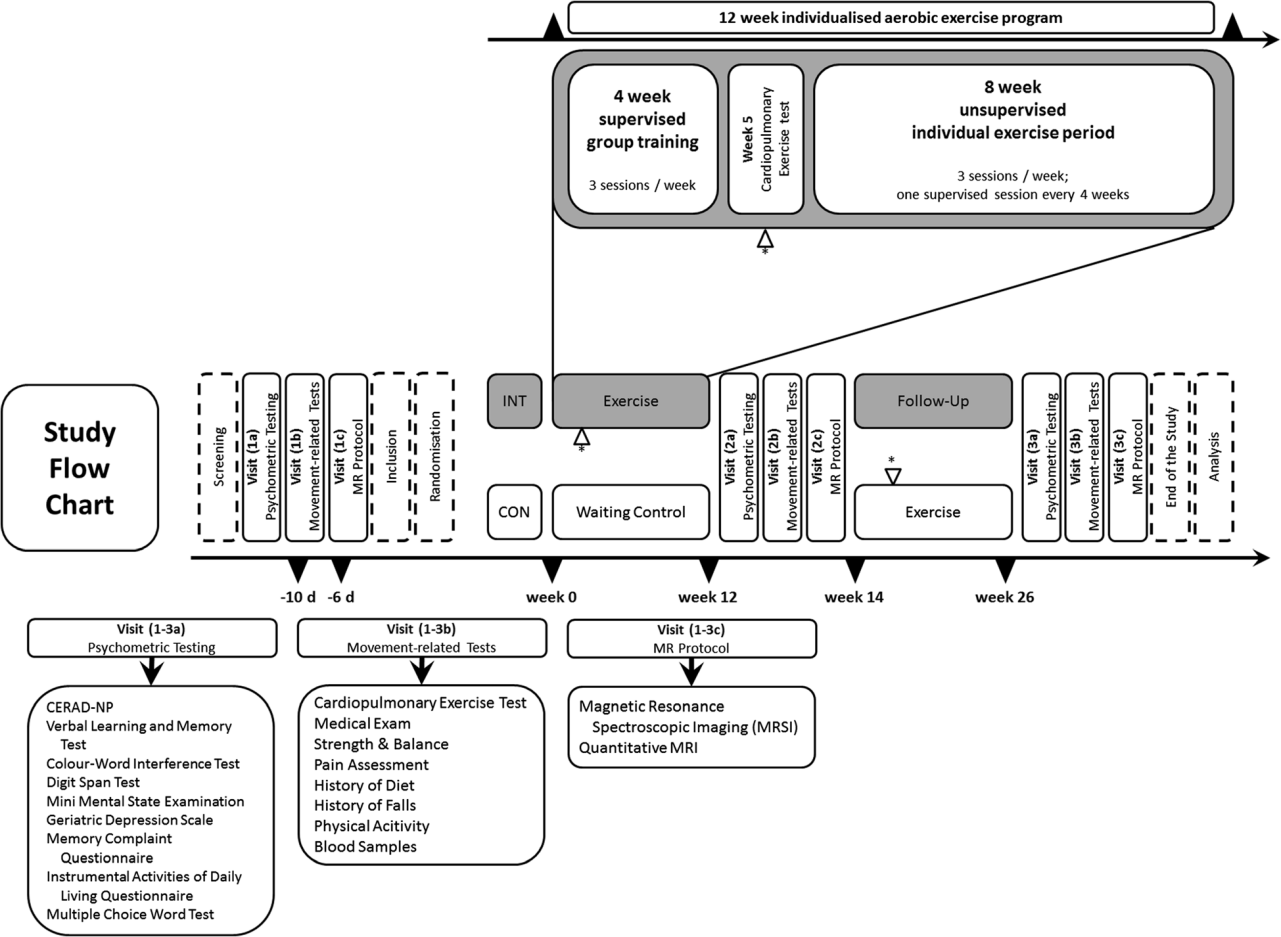
Study design. The figure details the dates in which participants are assessed or receive intervention. After screening at baseline, participants pass three visits (Visits 1a-c) at the respective departments; that is, the Institute of General Practice for psychometric testing, the Department of Sports Medicine for movement-related testing and the Institute of Neuroradiology for the conduction of the magnetic resonance (MR) protocol. Participants fulfilling all inclusion criteria are than randomly allocated to two groups: the intervention group (INT) or the waiting control group (CON). In the INT group, participants start a 12-week individualised aerobic exercise programme on a bicycle ergometer whereas in the CON group they continue their used daily activity for another 12 weeks. After 12 weeks, participants are reassessed at the above mentioned departments (Visits 2a-c). Participants in the CON group can now decide to perform the exercise programme too. Follow-up ends at 24 weeks (Visits 3a-c).

Main outcome is the change in cerebral metabolism, assessed by Magnetic Resonance Spectroscopic Imaging (MRSI). Analysis of all records is performed by blinded evaluators. The total follow-up period per participant is 6 months (see Figure 1).

### Setting

Recruitment of participants and implementation of the intervention takes place within the setting of three assisted living facilities in Frankfurt am Main, Germany, all together offering residency to more than 300 people. In addition, the press agency of the university released information to local print media on a single occasion. Besides common supportive facilities, the living residences provide a regular programme for different leisure activities for their residents, including cultural activities, lectures and sport/physical activities.

Participants likely for inclusion are screened by the Institute of General Practice, Goethe University, Frankfurt am Main, Germany. Written informed consent is obtained. Baseline cognitive data is assessed by means of questionnaires and psychological testing (Visit 1a, Figure 1). Subsequently, participants are grouped into blocks of five people and scheduled for a visit at the Department of Sports Medicine (Visit 1b, Figure 1), where they pass a thorough medical check-up, to screen for clinically significant health conditions. At Visit 1b we also assess movement-related parameters; that is strength and posture, specific questionnaires (for example, nutrition, history of every day physical activity, history of pain) and a cardiopulmonary exercise test (CPET). Five days later, the same group undergoes brain scans at the Brain Imaging Centre Frankfurt, Germany, for the assessment of brain structure (grey and white matter volume) and brain metabolites (N-acetyl-aspartate (NAA), glutamine, glutamate, myo-inositol, creatine, phosphocreatine, adenosine triphosphate (ATP), adenosine diphosphate (ADP) (calculated based on the equilibrium constant of the creatine-kinase reaction), inorganic phosphate, phosphoethanolamine, glycerophosphoethanolamine, phosphocholine, and glycerophosphocholine; Visit 1c, Figure 1). Participants are randomised to either receive intervention during the subsequent 12 weeks (group INT) or after a waiting period of 12 weeks (group CON). If allocated to INT, participants start the individualised exercise intervention in the integrated gym hall of one of the participating residencies 6 days after the magnetic resonance imaging (MRI) scans. After 4 weeks, the CPET is repeated and exercise intensity adjusted accordingly. All participants are told not to change their habitual physical activity during the following 3 months, except for the intervention in group INT. Twelve weeks after allocation all participants are scheduled for Visits 2a-c adhering once again to fixed inter-assessment intervals. Exercise intervention starts at this point for participants in group CON. Participants in group INT can continue their exercise voluntarily. Twenty-four weeks after inclusion, both groups are assessed once again (Visits 3a-c), complying with defined timeframes between measures.

### Types of participants

Only cognitively healthy participants are included in the study. Here, ‘cognitively healthy’ is defined as presenting no signs of dementia in cognitive performance during neuropsychological assessment and no impairment in activities of daily living. For inclusion participants must meet the following criteria: 1) aged 65 years or above, 2) show voluntariness, 3) have capacity to consent, 4) having passed a medical entry exam by the Department of Sports Medicine, 5) having given written informed consent. Participants presenting with any of the following exclusion criteria may not be included in the trial: 1) untreated clotting disorders; 2) musculoskeletal diseases significantly reducing mobility; 3) severe bacterial or viral infections; 4) severe respiratory diseases (Gold IV); 5) acute pulmonary embolism; 6) unstable angina pectoris or severe heart failure (New York Heart Association (NYHA) III or IV); 7) severe vascular disease of the extremities or the brain; 8) severe cardiopulmonary dysfunction; 9) acute myocardial infarction or early phase of rehabilitation: 10) critical aortic stenosis; 11) severe hypertrophic and obstructive cardiomyopathy; 12) untreated malignant arrhythmias; 13) untreated severe hypertension; 14) severe pulmonary hypertension15) symptomatic cardiac malformations (for example, septal defects, patent ductus arteriosus or valvular stenosis); 16) atrioventricular (AV)-block grade II or III; 17) left bundle-branch block; 18) complex ventricular arrhythmias; 19) cognitive impairment reflected in a score < 27 in a dementia screening test (Mini Mental State Examination) [22]; 20) specific exclusion criteria regarding MRI scans.

### Randomisation

The randomisation is performed on a 1:1 basis. If participants comply with inclusion criteria, the exercise physiologist opens a sealed and opaque envelope (compiled by an independent third party), allocating participants to either INT or CON. The randomisation sequence is generated using a computer-based algorithm (Research Randomiser, Version 4.0). For organisational reasons, the exercise physiologist schedules participants into groups of five. Participants within these blocks belong to the same treatment modality (that is intervention or control). Grouping is only allowed in the order of recruitment to the study. All assessors and study personal other than exercise physiologists are blinded. The same physiologist schedules upcoming visits. Participants are instructed not to communicate their random assignment to other assessors within the study. Organisational reasons for building groups of five include: a) facilitation of comparable timeframes between assessments (for example, sports medicine and brain imaging), visits (for example, Visit 1a and exercise (for example, from last assessment (MRSI) to the first exercise session or from the last exercise session to the beginning of the next assessment), and b) to conduct the exercise intervention in familiar groups of five participants.

### Intervention

#### Individualised aerobic exercise training

Participants in the INT group exercise 3 times a week for 30 minutes on a bicycle ergometer (optibike med, ergoline GmbH, Bitz, Germany) in the integrated gym hall of one of the participating residencies. Training is individualised as respective performance is adapted to the power at the first ventilator threshold (assessed during the CPET). The respective training intensity is stored on a chip card, which, when inserted into the ergometer, automatically sets the intensity and records training data (flash card system). During the first 4 weeks of intervention 2 of the 3 weekly training sessions are offered as group training supervised by the respective qualified exercise physiologist of the Department of Sports Medicine (group size: 5 participants). After 4 weeks, participants’ physical performance is reassessed at the Department of Sports Medicine. If necessary, workload is readjusted to achieve the initially defined exercise intensity. Starting the fifth week, only one session every 4 weeks is supervised, otherwise participants exercise unsupervised. After the 12-week exercise period participants can continue exercising voluntarily for the next 12 weeks.

For the whole duration of the study, all exercise performed by the participants on the ergometer is individually and automatically recorded on the respective chip card.

#### Waiting control

Participants of CON continue their regular physical activity behaviour for 12 weeks. They start the same exercise intervention after a reassessment at Visit 2 (see Figure 1) as INT.

### Outcome measures

At all time points, outcome parameters will be assessed within a timeframe of 1 week. At baseline it is the week immediately prior to inclusion into the study; at 12 and 24 weeks it is the week immediately following thereafter.

Main outcome measure is the change in cerebral metabolism assessed by MRSI. This method allows to display several parameters reflecting metabolic changes in the central nervous system. Therefore, we established three hypotheses, each to be tested individually:

- Primary hypothesis is that aerobic exercise leads to an increase of cerebral NAA mediated by plasma neurotrophins
- Secondary hypothesis stipulates an increase of markers of neuronal energy reserve: that is the ratio of phosphocreatine to creatine and of ATP to ADP
- Third hypothesis is an increase in the volume of cortical grey matter.

Measures for cerebral metabolism include MRI data acquisition and venous blood sampling. The MRI scans take place at the Brain Imaging Centre, Frankfurt am Main, Germany. Data are acquired using a 3-Tesla whole body scanner (Magnetom Trio, Siemens Medical AG, Erlangen, Germany) optimised for examinations of the cranium.

#### MR protocol

The entire MR protocol consists of two parts. The MRSI part is performed using a double tuned ^1^H/^31^P volume head coil (Rapid Biomedical, Würzburg, Germany) while the quantitative magnet resonance imaging (qMRI) data are acquired with an 8-channel array head coil (Siemens Medical, Erlangen, Germany). A three-dimensional T1-weighted image acquired during the MRSI part (2.5 minutes for acquisition) is used to coregister the MRSI to the qMRI data.

The MRSI protocol is planned on T2-weighted (T2-w) images in 3 orientations. For ^1^H MRSI a transversal slice (240 × 240 mm^2^ field-of-view (FOV), 16 × 16 matrix, 12 mm thickness, circular weighted acquisition scheme with 2 acquisitions at centre of k-space, 1,500 ms repetition time (TR), 30 ms echo-time (TE)) is recorded within a measurement time of 5 minutes. The volume of interest is selected by a combination of point-resolved selective spectroscopy and outer volume suppression.

For ^31^P MRSI, a three-dimensional MRSI slab (240 × 240 × 200 mm^3^ FOV, 8 × 8 × 8 matrix, circular weighted acquisition scheme with 10 acquisitions at the centre of k-space, 2 s repetition time, 2,000 ms TR, 60° pulses, 2.3 ms delay between excitation and recording of free-induction decay (FID), 12-minute measurement time)

Finally, the hippocampus contralateral to the dominant hemisphere is recorded with single voxel (SVS) ^1^H magnetic resonance spectroscopy (MRS providing concentration values for NAA, myo-inositol, glutamate, glutamine, the aggregated concentration of creatine and phosphocreatine, and the aggregated concentration of phosphocholine and glycerophosphocholine. A 15 × 20 × 30 mm^3^ voxel is recorded with Point Resolved Spectroscopy (PRESS) (TR 3 s, TE 30 ms, 96 acquisitions) in 5 minutes. The position of the voxel is planned on the three-dimensional T1-weighted data.

Before spatial Fourier transformation the matrix size of the MRSI data is doubled in all dimensions by zero filling. Within this process, the ^31^P slab is adjusted by grid-shifting to provide an ideal matching of ^31^P and ^1^H voxels; that is the ^1^H slice is positioned in the centre of a ^31^P slice and the in-plane ^31^P grid matches the ^1^H grid just exhibiting twice the scale.

qMRI of the brain is performed via T1 mapping based on the variable flip angle method [23]. In summary, two gradient echo data sets with different excitation angles are acquired with a FLASH-EPI readout to improve the signal-to-noise ratio (SNR) [24]. The acquisition parameters are: excitation angles 4°/24°, TR/TE = 16.4 ms/6.7 ms, matrix size 256 × 224 × 160, isotropic spatial resolution 1 mm, duration 9:48 minutes. B1 is measured according to [25], the duration of this measurement is 0:53 minutes. The T1 maps are corrected for B1 inhomogeneities and insufficient spoiling of transverse magnetisation [26]. Spatial non-uniformities of the receive RF coil are determined via a method that exploits the linear relationship between 1/PD and 1/T1 [27]. PD maps are derived by correcting the low angle data set for any T1, B1, and receive profile bias [27]. The total acquisition time of the qMRI protocol is 11 minutes.

#### Blood samples

Quantification of the neurotrophin BDNF is performed from venous blood samples in the Laboratory for Clinical Pharmacology, Psychiatric University Hospital Charité in Berlin using a modified fluorometric ELISA method as described previously [28].

Secondary outcome measures include psychometric testing and movement-related parameters.

#### Psychometric testing

*Psychometric testing* is performed at the Institute of General Practice and assesses the following cognitive functions: verbal declarative memory (Verbal Learning and Memory Test [29]; adapted German version of the Rey Auditory Verbal Learning Test [30]), frontal executive control (Colour-Word-Interference Test [31] adapted German version of the Stroop test [32] Trail-Making-Test Part B [33]) working memory (Digit Span Test forward and backward [34]) as well as semantic and phonematic fluency, nonverbal declarative memory and visual-constructive abilities by means of the CERAD-Plus (Consortium to Establish a Registry for Alzheimer’s Disease) Neuropsychological Battery [35]. In addition, the speed of cognitive processing is assessed by means of the Trail-Making-Test Part A [33]. Participants are screened for depressive symptoms with the Geriatric Depression Scale (GDS) [36]. Age-associated subjective memory impairment is assessed using a memory complaint questionnaire (MAC-Q [37]). Additionally, crystallised intelligence is assessed by means of a verbal intelligence test (Multiple-Choice Word Test: MWT-B, [38]).

Potential participants are screened for dementia and mild cognitive impairment using the Mini Mental State Examination [22] and the Instrumental Activities of Daily Living Questionnaire [39].

#### Movement-related parameters

*Movement-related parameters* are assessed at the Department of Sports Medicine including different parameters:

- Basic parameters:

assessment of vital parameters, body weight and height, anthropometry (Body Impedance Analyzer Nutriguard MS, Data Input, Pöcking, Germany) and waist-hip ratio.

b) Cardiopulmonary exercise test:

aerobic exercise capacity is determined by a physician-supervised CPET, a safe and effective method to assess functional capacity [40]. Participants are asked to refrain from alcohol or caffeine use and strenuous physical activity for 24 hours, from light physical activity for 4 hours, and from eating 2 hours before CPET. Body weight and height in light clothing are measured using standard techniques and calibrated equipment. During a graded exercise test on an electrically braked cycle ergometer (custo control, customed GmbH, Munich, Germany) the initial workload of 0 Watt is increased by 25 Watt every 3 minutes until exhaustion. Heart rate, electrocardiogram (ECG) data and ventilator data (breath-by-breath, open-circuit indirect spirometry (Cortex Metalizer 3B, Leipzig, Germany)) are registered continuously. Stringent quality protocols are followed to control for activities, mechanical and environment-related influences like relative humidity, mask fitting, sampling lines. Before each test the metabolic cart is calibrated using standard ventilatory volumes (0.2 and 3 l air/minute) and gases (outside air and 5% CO_2_, 16% O_2_) after sufficient warm-up. Ratings of perceived exertion (RPE; Borg-scale) and lactate concentrations (Lactate Scout+, EKF Diagnostics, Magdeburg, Germany) are measured at the end of each stage and after CPET termination. Participants are strongly encouraged throughout the test. Criteria for test cessation are volitional exhaustion (cadence below 60 rev/minute) or symptom limitation. For data analysis, peak oxygen uptake (VO_2peak_) is defined as the highest of all 30-s averages elicited during CPET [41]. First ventilatory threshold (VT1) was defined as: 1) non-linear increase in VCO_2_ versus VO_2_; 2) first non-linear increase of ventilation versus workload (V_E_/WL); 3) first increase of expiratory partial pressure of oxygen versus workload (P_ET_O_2_/WL); 4) first non-linear increase of ventilatory equivalent of oxygen (V_E_/VO_2_) versus workload with no concomitant increase of equivalent of carbon dioxide (V_E_/VCO_2_) [42,43].

c) Balance and strength [44]:

For postural sway (balance) and gait data acquisition, the capacitive force-measuring platform (30 Hz) WinFDM v0.0.41® (Zebris© GmbH, Isny, Germany) is used. Postural sway is estimated by the area of the 95% confidence ellipse calculated by using the maximum medial/lateral and anterior/posterior excursion of the centre of pressure (COP). For the measurement, we ask participants to stand upright (feet shoulder-width apart) as still as possible for 3 intervals of 90 s with a 2-minute rest interval in-between with arms folded across their chest and eyes covered. Gait speed (distance per time between subsequent floor contacts) is averaged on the basis of 10 individual strides monitored in the middle of a 10-m walkway when participants are required to walk across the sensor platform in a self-determined (usual) free-walking speed. Acceptable test-retest reliability has been described for this approach [45].

To obtain maximum isometric voluntary force (MIVF), the m3 (multi-muscle machine) Diagnos + © (Schnell© Trainingsgeräte GmbH, Peutenhausen, Germany) is used. The MIVF is obtained from the knee flexors/extensors of a randomly selected leg (a computer-compiled randomisation list defining right and left leg had been prepared prior to the experiment; predefined m3 angle for knee extensors = 120°/for knee flexors = °) and the lumbar muscles (extensors = 120°/for lumbar flexors = ). All subsequent measures of the knee muscles are performed on the random leg determined at Visit 1. All measures are performed in a respective standardised seated position, all participants are held fixed with a belt to the seat to avoid auxiliary muscle contraction. Three tests per muscle are performed with contractions lasting 5 s, separated by 2-minute rest intervals. Force × time is displayed on a screen providing an immediate feedback. In addition, participants are verbally encouraged in a standardised order to elicit maximal effort. Sufficient test-retest reliability and construct validity has been shown for this test [46].

d) Pain assessment:

German Pain Questionnaire (baseline and follow-up version; [47]), which includes the assessment of quantitative and qualitative pain description, pain intensity, disability and impairment, causalities and attributions, mental well-being, anxiety and depression, comorbidities, pretreatments and medications. The questionnaire consists of additional sections for optional use, assessing the quality of life impairment by pain inventory, quality of life and social law issues. Special follow-up forms are included that allow the assessment of the items at upcoming visits [47].

e) Dietary history:

Food frequency questionnaire DEGS1 assessing the nutrition of 53 different types of food over the last 4 weeks [48]. The questionnaire is only assessed at baseline.

f) History and fear of falling:

German version of the Falls-Efficacy-Scale International Version (FES-I) [49].

g) Physical activity:

As indicated by surveying the present and past history. In addition, accelerometry objectively reflects physical activity at baseline.

- Physical activity at present, assessing the last 7 days by means of the International Physical Activity Questionnaire (IPAQ) [50].
- Lifetime patterns of total physical activity including occupational, household, commuting and exercise/sports activities are assessed with a translated combination of the Lifetime Total Physical Activity Questionnaire [51], Historical Leisure Activity Questionnaire [52] and Retrospective Physical Activity Survey [53].
- Accelerometry (GT3X v4.4.0, ActiGraph, Pensacola FL, USA) assessing the average physical activity of 4 valid (at least 10 hours wear time) out of 7 consecutive days, at baseline [54].

### Data analysis and power calculations

Power calculations have been estimated on data published by Pajonk *et al*. [55], who showed a 3-month exercise programme to change the ratio of hippocampal NAA to total creatinetCr by 15% (n = 3 × 8). In our group, longitudinal changes of this effect size have been shown previously in groups larger than 15 subjects. Given poor data quality and a drop-out ratio of included subjects, both at 25%, into account, we estimated a group size of 30 participants to be adequate. Thus, the total sample size is 60.

Statistical analysis is performed according to current standards in reporting clinical trials differentiating for parametric and non-parametric data first and applying the respective tests thereafter. Changes over time are analysed applying repeated measures methods. If statistically different at baseline, secondary outcomes are introduced as possible confounders by means of covariate analysis.

#### MRSI data analysis

The ^1^H MRSI spectra are fitted with the commercially available software tool LCModel (downloadable test version at: http://s-provencher.com/pages/lcmodel.shtml; [56]), which simulates the spectra with a linear combination of model spectra and is considered to be the most suitable tool for the analysis of short-TE spectra [57]. Baseline correction is performed including macromolecules. The ^31^P data is analysed with the tool jMRUI [58], which was found to be more appropriate for these types of spectra.

#### qMRI data analysis

From the quantitative maps of PD and T1, it is possible to calculate synthetic anatomical data sets, showing the same contrasts as data sets acquired with the respective standard sequences. The advantage is that synthetic data sets do not suffer from any RF coil bias. Furthermore, purely T1-weighted data sets can be calculated by omitting the inclusion of PD, thus enhancing the contrasts. In the present case, MPRAGE [59] data sets are calculated, using a mathematical formalism described in the literature [60], assuming the parameters TR = 2,420 ms, TI = 960 ms, excitation angle 9°.

Changes in grey matter volume following the intervention are analysed with voxel-based morphometry (VBM) [61] in SPM8 (Wellcome Department of Cognitive Neurology, London, UK) running under Matlab 8 (Mathworks, Sherborn, MA, USA). Data preprocessing includes segmentation, spatial smoothing with a Gaussian Kernel of 10 mm full width at half maximum (FWHM) and spatial normalisation using the DARTEL toolbox (SPM8). The whole brain analysis is followed by a Region of Interest (ROI) Analysis. To allow the detection of alterations in hippocampal morphology, a VBM analysis using the hippocampus as ROI is performed. The hippocampus ROI is derived from the Wake Forest University (WFU) PickAtlas toolbox. Small volume correction (SVC) is used to reduce the number of comparisons being performed, increasing the chance of significant results in the ROI.

#### Psychometric data analysis

Neuropsychological test scores are used to calculate domain specific measures. Six cognitive domains are assessed from 8 tests: Executive functioning (Colour-Word Interference Test, Trail-Making Test Part B), Working memory (Digit Span Test forwards and backwards), Memory (Verbal Learning and Memory Test, CERAD figure recall), Visuospatial performance (CERAD figure drawing), Language (CERAD phonematic fluency, CERAD semantic fluency) and speed of cognitive processing (Trail-Making-Test Part A). Individual test scores are converted to z-scores using the mean and standard deviation of the entire sample. Similar to the approach by Vemuri *et al*. [62] the individual z-scores are averaged to create six domain scores. The six domain scores are averaged to calculate a global cognitive summary score.

#### Balance and MIVF data analysis

The average of the 3 posture measurements (areas of the 95% confidence ellipse) is used for data analysis.

Collected data is analysed by Diagnos 2000 (Trainsoft© GmbH, Moorenweis, Germany). The highest value of the three trials (randomised leg selection) (N m kg^−1^) is considered to be representative of MIVF and is used for statistical analysis.

## Discussion

This study explores the effects of a supervised aerobic exercise intervention on cerebral metabolism as a correlate of cognitive functioning in cognitively healthy older adults. It is a randomised controlled intervention study comparing aerobic exercise on an ergometer in older people with a waiting control group. Beside severe restraints in patients’ lives, cognitive impairment is associated with higher mortality and lower functional recovery [63]. Several cofactors account for this condition [19,20]. In some cases, memory complaints have been described uncoupled from dementia as expressions of low mood and impairments in activities of daily living (for review: [64]). The strength of the present protocol is to control for a large subset of possibly influencing factors, in particular history of physical activity, diet and pain. This comprehensive understanding allows us to identify or control for confounders predicting improved or reduced benefit from physical exercise in older people. Still, multiple measures can be concerned a limitation when interpreting the results of a study. In the present study, we accentuate on the primary outcome (changes in cerebral metabolism) and will strictly adhere to statistical conventions with the analysis of secondary outcomes.

Participants exercise at intensities derived from an individually measured and consecutively readjusted submaximal physiologic threshold. This threshold concept is regarded as a standard in cardiopulmonary exercise testing in public health recommendations [65] and allows valid and comparable intensity determination even if maximal workload (VO_2max_) is not attainable.

This is the first study assessing the influence of ergometer exercise at individually established and readjusted intensities on brain metabolism in cognitively healthy older adults. Yet, a dose-dependency between physical exercise and cognitive performance in older adults in neuroprotection has been suggested [66]. Both higher levels of fitness [67] and a higher estimated VO_2max_ [2], have been shown to be favourable regarding cognitive performance in older people. A meta-analyses including 16 prospective studies with 3,219 patients at follow-up showed the relative risk for dementia to be reduced by 28% when comparing the highest to the lowest physical activity category [68]. Still, the optimal exercise dosage and type of exercise remain unclear.

Beside aerobic training, the effects of resistance, cognitive and novel dual-task exercise training interventions for the preservation or improvement of cognitive health have proven to be effective, well-tolerated and safe for older adults (for review: [69]). However, their potential in the improvement of cognitive impairment seems to be maximised when coupling individualised or progressive, moderate-to-high aerobic-based exercise with dual-task training over a period of 1 to 12 months. Effects of resistance training (RT) programmes are promising, but current evidence is inconclusive supporting the effectiveness of RT as a stand-alone treatment (for review: [69]). Accordingly, we decided to investigate the effects of a fully individualised, structured aerobic exercise intervention, with a training intensity that is adjusted to participants’ performance for a period of 12 weeks, within a 24-week follow-up for preserving or enhancing cognitive performance in older adults [70]. The regimen is conducted in groups of five participants each. Trainings take place in the familiar setting of the living facilities.

Compared to laboratory animals the neuronal mechanisms underlying the positive influence of physical activity on cognition in humans are far less understood. Previously, neuroimaging has been used to investigate the effect of regular aerobic exercise on brain structure and function in older healthy adults *in vivo*. Structural MRI (sMRI) studies demonstrate an exercise-induced increase of grey matter volume in several brain regions, particularly within the hippocampus and prefrontal cortex [13,71,72], thereby counteracting an age-associated atrophy in these regions. The observed brain changes were positively correlated with improvement in cognitive function and with an increase in serum BDNF [13,72]. Studies using functional MRI (fMRI) could show that exercise increases functional connectivity in higher-level cognitive networks, thereby improving executive function [73,74]. Although these studies support a positive effect of physical activity on structural and functional cerebral plasticity in healthy ageing they do not contribute to the clarification of metabolic pathways underlying the observed cognitive improvement. In contrast to sMRI and fMRI, information on brain metabolism can be gathered by two complementary and fully quantitative neuroimaging methods *in vivo*: MRS and positron emission tomography (PET). ^31^P MRS is the ‘gold standard’ technique allowing absolute quantification of metabolites closely related to cerebral energy metabolism (ATP, ADP, phosphocreatine, creatine), while ^1^H MRS provides information on neuronal viability/integrity (via the neuronal marker NAA) in different brain regions. On the other hand, PET with [^18^ F]fluoro-deoxy-glucose ([^18^ F]FDG) is the ‘gold standard’ technique for estimation of glucose turnover providing quantitative assessments of the (regional) metabolic rate of glucose metabolism.

Although the feasibility of using MRS and FDG-PET for studying brain metabolism in ageing and neurodegenerative disorders has been demonstrated in numerous studies there is a paucity of studies using these methods to investigate regional metabolic changes induced by physical exercise. To our knowledge, only one recently published study addressed the relationship between aerobic fitness, cognition, and the regional concentrations of MRSI-derived metabolites in the brains of older people [75]. This study found a positive association between aerobic fitness and NAA in the frontal lobe indicating an influence of physical fitness on regional neuronal viability and/or density. However, this was a cross-sectional study which precludes a sound conclusion on the causal relationship between exercise and brain metabolism.

The current study aims to shed light on the influence of physical exercise on brain metabolism in healthy ageing. Based on the findings mentioned above the main hypothesis under investigation is that regular physical exercise leads to an enhancement of cerebral energy metabolism in the brains of older people that is closely related to an increase in the concentration of metabolic markers for neuronal viability and density. We assume that these changes occur particularly in the hippocampus, are mediated by a release of the neurotrophin BDNF, and predict an improvement of cognitive function including memory and executive control. Specifically, we are interested in the short- and mid-term cerebral metabolic and cognitive effects of a supervised individualised exercise intervention assuming that regular physical activity has the potential to counteract brain ageing.

## Trial status

At the time of manuscript submission, 50 participants have been included into the study with no participant having completed. Study completion is expected to be September 2015.

## Abbreviations

ADP: adenosine diphosphate
ATP: adenosine triphosphate
AV: atrioventricular
BDNF: brain-derived neurotrophic factor
CERAD: Consortium to Establish a Registry of Alzheimer’s Disease
COP: centre of pressure
CPET: cardiopulmonary exercise test
ECG: electrocardiogram
ELISA: enzyme-linked immunosorbent assay
FDG: ^18^fluoro-deoxy-glucose
FES-I: Falls-Efficacy-Scale International Version
FID: free-induction decay
fMRI: functional MRI
FWHM: full width at half maximum
GDS: Geriatric Depression Scale
IGF-1: insulin-like-growth factor 1
IPAQ: Activity Questionnaire
MAC-Q: memory complaint questionnaire
MIVF: maximum isometric voluntary force
MRI: magnet resonance imaging
MRS: magnetic resonance spectroscopy
MRSI: Magnet Resonance Spectroscopic Imaging
NAA: N-acetyl-aspartate
NYHA: New York Heart Association
PET: positron emission tomography
PRESS: Point Resolved Spectroscopy
qMRI: quantitative magnet resonance imaging
ROI: Region of Interest
RPE: ratings of perceived exertion
sMRI: structural MRI
SNR: signal-to-noise ratio
SVC: small volume correction
SVS: single voxel spectroscopy
TE: echo-time
TR: repetition time
VBM: voxel-based morphometry
VO_2peak_: peak oxygen uptake
VT1: first ventilatory threshold
WFU: Wake Forest University
